# Analytical Performance of Next-Generation Sequencing and RT-PCR on Formalin-Fixed Paraffin-Embedded Tumor Tissues for *PIK3CA* Testing in HR+/HER2− Breast Cancer

**DOI:** 10.3390/cells11223545

**Published:** 2022-11-09

**Authors:** Konstantinos Venetis, Francesco Pepe, Elisabetta Munzone, Elham Sajjadi, Gianluca Russo, Pasquale Pisapia, Mariia Ivanova, Giuseppina Bonizzi, Davide Vacirca, Alessandra Rappa, Alberto Ranghiero, Sergio Vincenzo Taormina, Giuseppe Viale, Giancarlo Troncone, Massimo Barberis, Elena Guerini-Rocco, Umberto Malapelle, Nicola Fusco

**Affiliations:** 1 Division of Pathology, IEO, European Institute of Oncology IRCCS, Via Giuseppe Ripamonti 435, 20141 Milan, Italy; 2 Department of Oncology and Hemato-Oncology, University of Milan, Via Festa del Perdono 7, 20122 Milan, Italy; 3 Department of Public Health, Federico II University of Naples, Via S. Pansini, 5, 80131 Naples, Italy; 4 Division of Medical Senology, IEO, European Institute of Oncology IRCCS, Via Giuseppe Ripamonti 435, 20141 Milan, Italy

**Keywords:** breast cancer, biomarkers, *PIK3CA*, NGS, RT-PCR

## Abstract

Somatic mutations in *PIK3CA* are present in ~40% breast cancers (BC); their detection in hormone receptor (HR)+/HER2− tumors allows for selecting patients with advanced disease eligible for *PIK3CA* targeting with alpelisib. The choice of what type of *PIK3CA* testing approach to adopt and which tissue sample to analyze is a new task in breast pathology. In this methodological study, we sought to assess the performance of next-generation sequencing (NGS) and RT-PCR for *PIK3CA* testing on archival formalin-fixed paraffin-embedded (FFPE) primary tumors and corresponding metastases. Sixteen HR+/HER2− BC with known *PIK3CA*-mutated status (ex. 7, 9, and 20) on metastatic samples by means of amplicon-based targeted NGS were selected, and *n* = 13 of these samples were re-tested with a commercially available CE-IVD RT-PCR assay. All available primary tumors (*n* = 8) were tested with both methods. NGS detected mutations in all samples, while RT-PCR in *n* = 2 sample-pairs and overall, in *n* = 5/8 (62.5%) primary tumors and 7/13 (53.8%) metastases (κ = 0.09; 95% CI, −0.69–0.87). Slight agreement (κ = 0; 95% CI, −0.59–0.59) was observed between NGS and RT-PCR, with the former being generally more sensitive in cases with low DNA quality and quantity. Post hoc visual inspection of the RT-PCR data increased the concordance to 76.9%. Targeted NGS offers reliable and robust *PIK3CA* testing on both tumor and metastasis FFPE samples; the accuracy of RT-PCR depends on the DNA quantity and quality. In HR+/HER2− BC, both the selection of the *PIK3CA* testing strategy of FFPE tissues and which sample to analyze should consider several technical parameters and should be tailored for each case.

## 1. Introduction

Breast cancer is the most prevalent type of tumor and the primary cause of cancer-related deaths among women globally [1]. Approximately two-thirds of these tumors express hormone receptors (HR) and lack HER2 overexpression and/or amplification [2,3]. For these patients, endocrine therapy (ET) combined with cyclin-dependent kinases (CDK)4/6 inhibitors is the gold standard combination in the metastatic setting [4,5]. A remarkable proportion of patients, regrettably, might experience ET resistance for several reasons, including the upregulation of phosphoinositide 3 kinase (PI3K)/Akt/mammalian target of rapamycin (mTOR) signaling [6,7,8]. Activating mutations in *PIK3CA*, the gene encoding the p110α subunit of PI3K, occur in ~40% of breast cancers, and they are driver events for tumorigenesis and tumor progression [9,10,11,12]. The SOLAR-1 study was a phase III randomized trial investigating the efficacy of the α-selective PI3K inhibitor and degrader alpelisib (BYL719; Novartis Pharma AG) plus fulvestrant in patients with HR+/HER2− advanced breast cancer who had received prior ET [2,13,14]. Significant clinical benefits were observed in the *PIK3CA*-mutated cohort compared to the control group. These results led to the clearance of this drug for the treatment of patients with HR+/HER2− *PIK3CA*-mutated advanced breast cancer by both the U.S. Food and Drug Administration (FDA) and European Medicines Agency (EMA) [15].

The approval of alpelisib was accompanied by an RT-PCR-based companion diagnostic test for *PIK3CA* status assessment (therascreen *PIK3CA* RGQ PCR Kit CE-IVD, Qiagen) [16,17]. In addition to the SOLAR-1, another study (i.e., BYLieve) showed that *PIK3CA* testing can be carried out on tumor tissue specimens (primary tumor or metastasis) and/or in circulating tumor DNA (ctDNA) [14,18]. Given that this type of mutational analysis is new in breast cancer predictive pathology, adopting the most appropriate diagnostic strategy in the real-world clinical practice is not trivial [19,20]. In this scenario, the issues related to tissue availability in biopsy specimens and the improved knowledge of the mutational landscape of breast cancer encouraged the widespread diffusion of next-generation sequencing (NGS) platforms in the diagnostic routine [16,21,22]. However, the differences in the analytical performance of NGS and RT-PCR, the selection of the most appropriate tissue sample (i.e., primary site vs. metastasis), and the stability of *PIK3CA* mutations over the course of the disease have not been well documented so far in breast cancer.

Formalin-fixed paraffin-embedded (FFPE) tumor tissues represent the most employed biosources for molecular testing in pathology laboratories worldwide [23]. Therefore, the optimization of *PIK3CA* molecular analysis in breast cancer FFPE samples, either primary or metastasis, is a prerequisite for the implementation of this test and its integration with liquid biopsies. This study aims to investigate the concordance rate of NGS and RT-PCR in detecting *PIK3CA* mutations and to evaluate whether primary tumor samples would be able to recapitulate the mutational status of the metastases.

## 2. Materials and Methods

### 2.1. Study Design

**Figure 1:**
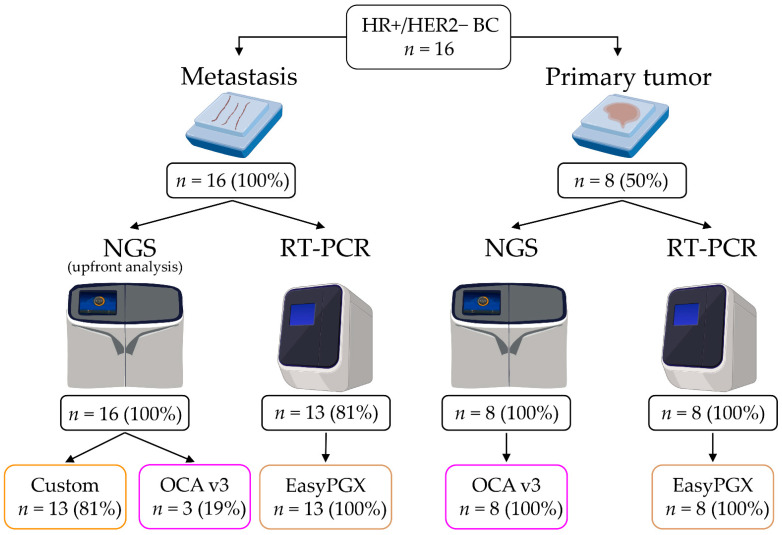
Study design. HR, hormone receptor; HER2, human epidermal growth factor receptor 2; BC, breast cancer; NGS, next-generation sequencing; Custom, custom panel (targeted sequencing); OCA v3, Oncomine Comprehensive Assay v3 (Thermo Fisher Scientific); EasyPGX, RT-PCR semi-closed assay (Diatech Pharmacogenetics).

This study was approved by the local Ethics Committee under approval number #UID3472; written informed consent was obtained from patients for use of tissue samples. According to the European Union’s General Data Protection Regulation (GDPR), all information regarding the recruited patients was pseudoanonymized [24]; the samples were handled in compliance with the Helsinki Declaration (http://www.wma.net/en/30publications/10policies/b3/, accessed on 14 September 2022). The study was designed to test the *PIK3CA* mutational status on different types of samples using different techniques, as depicted in Figure 1. The predictivity of *PIK3CA* mutations has been previously demonstrated [2] and was out of the scope of the present investigation.

**Figure 2:**
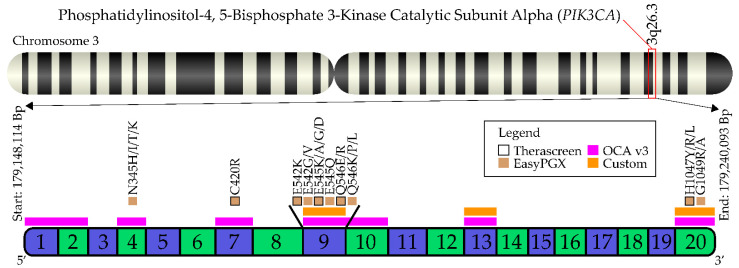
Exon structure of *PIK3CA* gene with reference range of the assays employed in this study and the therascreen *PIK3CA* RGQ PCR Kit test employed in the SOLAR-1 trial. EasyPGX, EasyPGX *PIK3CA* testing, Diatech Pharmacogenetics; NGS, next-generation sequencing; OCA, Oncomine™ Comprehensive Assay v3M, ThermoFisher Scientific; Custom, custom targeted panel, ThermoFisher Scientific, Waltham, MA, USA.

From the institutional pathology archives of the IEO, European Institute of Oncology IRCCS, Milan, Italy a total of *n* = 16 *PIK3CA*-mutant HR+/HER2− metastatic breast cancer patients were retrospectively selected. The *PIK3CA* status of the metastatic samples was available for all patients because it was routinely assessed by NGS using either a custom panel or the OCA v3 (Oncomine Comprehensive Assay v3), both Thermo Fisher Scientific (Waltham, MA, USA) (Figure 2). All cases with residual material (*n* = 13) for further analyses were re-tested with an RT-PCR semi-closed assay (EasyPGX, Diatech Pharmacogenetics, Ancona, Italy) covering 10 *PIK3CA* regions, including the 5 regions covered by the therascreen (Figure 2). The available corresponding primary tumors (*n* = 8) were subjected to *PIK3CA* testing with both NGS and RT-PCR.

### 2.2. Patients and Tissue Specimens

All patients were diagnosed and managed at the aforementioned institution between 2017 and 2021. Hematoxylin and eosin-stained serial sections of each case were centrally reviewed, re-classified, and re-graded according to the latest WHO recommendations and the Nottingham histologic grading system, respectively [25,26]. HR and HER2 were re-tested and re-analyzed according to the latest ASCO/CAP recommendations, with particular attention to identifying HR-low and HER2-low cases [27,28,29]. Pathologic re-staging was performed following the 8th edition of the American Joint Committee on Cancer (AJCC) *Cancer Staging Manual* [30].

### 2.3. Nucleic Acids Purification

Seven unstained slides at four-μm-thick sections from representative FFPE tissue blocks were used for the analyses. In 10 out of 24 samples (42%), manual microdissection was performed before nucleic acid isolation to enrich tumor cell content using a sterile scalpel. DNA was extracted using the Maxwell^®^ RSC DNA FFPE Kit (Promega, Madison, WI, USA) following the manufacturer’s instructions and then quantified by the QuantiFluor^®^ ONE dsDNA System (Promega) on the Quantus™ Fluorometer (Promega).

### 2.4. Next-Generation Sequencing (NGS) Analysis

For the custom NGS panel, library amplification was carried out on automatized Ion Chef system (ThermoFisher Scientific) according to manufacturer instructions. A total of 15 ng was dispensed on Ion Code plates and amplified with Ion AmpliSeq DL8 kit (ThermoFisher Scientific) following standardized thermal conditions (23 cycles for amplification step, 4 min each). Then, purified libraries were diluted at 30 pM and newly loaded into the Ion Chef instrument for automatic template preparation and loading chip. Finally, libraries were automatically loaded on an Ion 520™ Chip and sequenced on an Ion S5™ System (ThermoFisher Scientific) following manufacturer instructions. Data analysis was performed as follows: after alignment to the hg19 human reference genome, coverage analysis with custom bed-files was assessed on coverage plug-in (v.5.0.2.0) from Torrent Suite [v.5.0.2]; variant Caller plug-in was carried out with a dedicated workflow on Ion Reporter Torrent Suite 5.16. Molecular alterations found in *PIK3CA* with a minimum coverage depth of 500×, allele coverage and a quality score ≥ 20, and minimum variant frequency of 1% were annotated. For OCA assay, libraries were prepared by using an Ion AmpliSeq DL8 kit (ThermoFisher Scientific) on an Ion Chef system (ThermoFisher Scientific) following manufacturer instructions. After library reamplification and barcoding, libraries were diluted at 30 pM and newly loaded into the Ion Chef instrument for automatic template preparation and loading chip. Finally, the barcoded chip was sequenced on an Ion S5™ System (ThermoFisher Scientific) following manufacturer instructions. The sequence data analysis was carried out by using customized analysis parameters on Torrent Suite™ 5.16. Only *PIK3CA* single nucleotide variants with a minimum coverage depth of 500×, allele coverage and a quality score ≥ 20, and a minimum variant frequency of 1% were reported.

### 2.5. RT-PCR Analysis

RT-PCR was conducted using the EasyPGX^®^ ready *PIK3CA* kit (Diatech Pharmacogenetics) on the thermalcycler AriaDx Real-Time PCR System (Agilent, Santa Clara, CA, USA), according to the manufacturer’s instructions. This assay enables the detection of the most clinically relevant exons 9–20 *PIK3CA* alterations. An optimum of 15–30 ng/well is required to successfully perform the sequencing run. Data were analyzed using proprietary software (Agilent Aria software version 4.08) that automatically evaluates positive or negative results starting from the quantification cycle (Cq) and ΔCq values inspection.

### 2.6. Statistical Analyses

All statistical analyses were performed using SPSS software ver. 26.0 (IBM). The agreement between testing methods on the same biological sample and between different biospecimens using the same technical approach was evaluated with Cohen’s Kappa coefficient (κ).

## 3. Results

### 3.1. Clinicopathological Features

The median age at diagnosis of the patients included in this study was 52.5 years (range, 32–73 years; mean ± standard deviation (SD), 52.5 ± 11.2 years). Except for one case with lobular histology, all cases were invasive carcinomas of no special type. All cases were confirmed to be ER+, while eight patients had an ER+/PgR− tumor. Taken together, all of the cases included in this study at the time of the primary tumor diagnosis were HR+/HER2−. Among these, one patient developed an HER2+ disease after tumor progression. Furthermore, six (38.0%) HER2-negative cases displayed an HER2-low phenotype, i.e., immunohistochemistry (IHC) score 1+ or 2+ with no gene amplification. All other samples were HER2-zero. Among the metastatic sites, the liver was the most commonly affected (*n* = 8, 50.0%), while metastases were also observed in pleura (*n* = 2, 12.5%), iliac crest (*n* = 2, 12.5%), and distant lymph nodes (*n* = 2, 12.5%). Other metastatic sites included the pelvis and thorax (*n* = 1, 6.2%, respectively). Therapy with CDK4/6 inhibitors was administered to six (37.5%) patients. The clinicopathological characteristics of the patients included in this study are summarized in Table 1.

The time interval between disease diagnosis and metastasis in patients for whom both primary tumor and metastasis FFPE samples were available ranged from 38 to 127 months, as shown in Table 2.

### 3.2. Concordance between Primary Tumor and Metastasis Samples Using NGS

To define the mutational status of *PIK3CA* using NGS and to assess whether this can be recapitulated by the primary tissue, we analyzed both the primary tumor samples when available (*n* = 8, 50.0%) and all the metastases (*n* = 16, 100%). In the latter, NGS multigene panels detected *PIK3CA* ex. 7, 9, and 20 mutations. The mutations captured by this analysis include E453K (*n* = 1, 6.2%), E542K (*n* = 1, 6.2%), E545K (*n* = 3, 18.8%), H1047L (*n* = 1, 6.2%), H1047R (*n* = 6, 38%), N1044K (*n* = 1, 6.2%), N1068Kfs*5 (*n* = 1, 6.2%), Q546K (*n* = 1, 6.2%), and Q546R (*n* = 1, 6.2%). A high agreement between primary tumor samples and metastases analyzed with NGS and a concordance rate of 100% was observed regardless of samples’ technical characteristics and limitations (e.g., low DNA concentration, potential DNA fragmentation due to long-term archival tissues, and low tumor cell content). The overall results of this comparison and the specific characteristics of the samples are included in Table 3. This finding suggests the high reproducibility of NGS both in primary tumors and metastases. In addition, it suggests that the former, even if extensively stored, represents a valuable source for *PIK3CA* testing in the absence of metastasis samples.

### 3.3. Concordance between Primary Tumor and Metastasis Samples Using RT-PCR

Then, we asked whether the detection of *PIK3CA* alterations would be identifiable in primary tumor samples and metastases using RT-PCR assays. Based on the quantity and quality of the available material (i.e., archival slides and blocks, residual extracted DNA), all primary tumors (100%) and 13 out of 16 (81.3%) metastases were analyzed. Regarding the former, *PIK3CA* mutations were detected in five (62.5%) cases, while three (37.5%) cases were reported as wild-type, with two of them (66.6%) being out of the reference range of the assay (Table 4). By analyzing the metastasis samples, the RT-PCR assay managed to identify *PIK3CA* mutations in seven (53.8%) cases. In three (23.1%) of them reported as wild-type, two (66.6%) were not included in the reference range of the assay (Table 4). Moreover, the analysis software failed to provide results in three3 (23.1%) cases due to low DNA concentration. In these, despite the failure of the system in reporting the alteration, visual inspection of the raw data confirmed the presence of the mutation (Supplementary Figure S1). Overall, in three cases (i.e., PIK_004, PIK_007, and PIK_016), the RT-PCR assay identified mutations in the primary tumor, whereas these were not found in the corresponding metastases (i.e., failed or wild-type). Instead, in only one case, the mutation identified in the metastasis was not recapitulated by the primary tumor. The concordance rate between primary tumor and corresponding metastasis tested by RT-PCR was 40.0% (*n* = 2/5) with a Cohen kappa=0.09 (95% CI, −0.69–0.87), suggesting a slight agreement.

### 3.4. Comparison of NGS and RT-PCR

**Figure 3:**
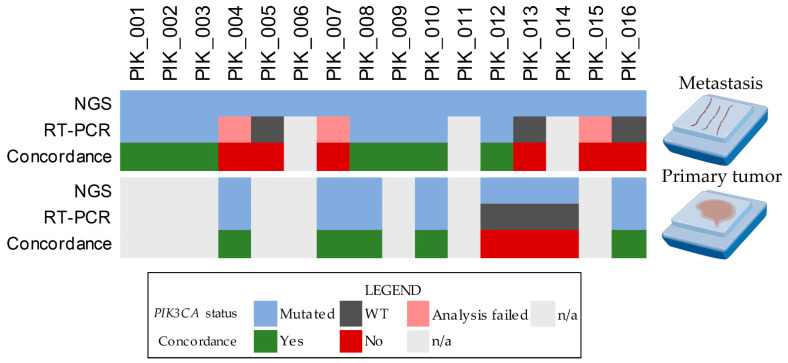
Heatmap illustrating the results of the analysis at a single-patient level for both primary tumors and metastasis samples. Each column represents a patient, and each row represents the type of assay with a concordance of both if applicable, color-coded according to the legend below. WT, wild-type; n/a, non-applicable.

We next compared the results obtained from each technology to evaluate their analytical performance and their capability of detecting *PIK3CA* mutations both in primary tumor and metastasis samples. Concerning the analysis of the primary tumors, RT-PCR verified the mutations identified by NGS in five cases, again confirming a slight agreement with a Cohen kappa = 0 (95% CI, −0.89–0.89) and an overall concordance rate of 62.5%. Regarding the metastasis samples, the results of the EasyPGX system were concordant with those of the NGS panels in 7 out of 13 cases (53.8%), and a slight agreement was observed, with a Cohen kappa = 0 (95% CI, −0.59–0.59). Among these, exons 9 and 20 of *PIK3CA* harbored three (23.1%) and four (30.7%) molecular alterations, respectively. When the raw data of RT-PCR analysis were visually inspected, *PIK3CA* hotspot mutations were observed in 3/13 (23.1%) additional samples, improving the concordance rate to 76.9%. The results of the overall comparison between the two testing methods in both sets of samples are summarized in Figure 3.

## 4. Discussion

This proof-of-principle study evaluated the concordance rate for *PIK3CA* assessment between breast cancer primary tumors and matched metastases and the consensus among NGS and RT-PCR for this type of molecular testing. Our analyses confirm the robustness of targeted NGS and the reliability of the RT-PCR for *PIK3CA* testing. Additionally, we showed the high analytical performance of NGS-based panels able to detect *PIK3CA* pathogenic mutations both in surgical resections and biopsy specimens. Furthermore, we demonstrated the moderate efficacy of the RT-PCR assay to confirm the *PIK3CA* alterations identified by NGS due to technical limitations of the former in the analysis of challenging samples. Given the substantial overlap of *PIK3CA* status in primary tumors and metastases, the choice of what sample to test in HR+/HER2− breast cancer should be primarily driven by the quality and quantity of FFPE material available, regardless of the anatomical site. However, the interval between the diagnosis of the primary tumor and the occurrence of the metastasis as well as the therapy administered to each patient should be considered.

*PIK3CA* molecular testing has rapidly emerged as a new clinical request at the sight of data from the SOLAR-1 study regarding the efficacy of alpelisib in combination with fulvestrant in patients with advanced HR+/HER2− breast cancer previously treated with endocrine therapy [2,14]. Despite the approval of alpelisib that the FDA approved alongside the Therascreen *PIK3CA* mutation assay as a companion diagnostic, the increasing number of clinically relevant molecular alterations able to select *PIK3CA*-mutated breast cancer patients escalates the need for highly performant molecular testing methods [10]. Thus, the identification of the most suitable testing strategy according to internal expertise and available biospecimens represents an opening challenge in each institution performing molecular tests [16]. In this context, primary tumor and metastasis samples feature distinct technical burdens that drastically impact the analytical performance of the currently available testing methods. The latter is usually characterized by low DNA concentration that derives from moderate tumor cell content [20,31]. Given the overall long-term survivorship and long time to disease progression in patients with HR+/HER2− breast cancers, primary tumor samples are frequently old and may potentially be characterized by a high level of DNA fragmentation [32]. Despite these hindrances, in our cohort, NGS approaches adopted for *PIK3CA* molecular analysis managed to successfully identify mutations in all cases demonstrating an absolute concordance between primary and metastasis samples. In a recent study, a droplet digital PCR (ddPCR)-based approach highlighted a slightly lower concordance rate of 87.8% between primary tumors and metastasis samples [33]. This could either be related to intratumor heterogeneity or the evolution of the disease [21,34]. Moreover, the timing of biopsy sampling can influence the detection rate of molecular alterations [35]. However, in the recently published analyses from the AURORA study which analyzed 381 primary tumors and matched metastasis samples, an enrichment of *PIK3CA* mutations in metastasis was observed [36]. Thus, it is relevant to have a higher number of *PIK3CA* mutations in progressive or recurrent diseases [37]. Taken together, both the selection of the most appropriate genomic assay and the biospecimen to be analyzed should be considered when *PIK3CA* molecular testing is carried out.

The majority of molecular biology laboratories can evaluate the mutational status of *PIK3CA* using RT-PCR-based assays [38]. Accordingly, we investigated the analytical performance of a commercially available RT-PCR assay to identify mutations detected by NGS panels. Overall, the mutational analysis of *PIK3CA* status in primary tumor and metastasis samples by RT-PCR showed a low concordance rate of 33.3%. Failures occurred irrespective of the sample type and specifically, in the metastases, they were mainly related to low DNA concentration (*n* = 2, 15.4%). Importantly, in both cases, the mutation was found in the primary tumor sample in which DNA concentration was higher (0.26 vs. 9.11 and 0.18 vs. 0.54 ng/μL, respectively). Therefore, RT-PCR assays should be chosen carefully based on their technical characteristics and should be used with caution in samples with low DNA concentration. In such cases, complementary tests on matched primary tumors, which are usually richer in terms of material, should not be excluded to avoid false negative results.

By comparing the analytical performance of the two platforms both in primary tumors and matched metastases, we found discrepancies that need to be contextualized to better establish the advantages and limitations of each method. In the metastases, the moderate concordance between NGS and RT-PCR (overall concordance rate 53.8%) was entirely associated with failures in the analyses of the latter. In 4/6 (66.6%) of the discordant cases, which had low DNA concentration, this test either failed to detect the *PIK3CA* mutation or reported a false negative result, highlighting again that the quantity of nucleic acids drastically impacts the successful detection of genetic alterations. The lack of consensus in the other two cases (33.3%) was due to the limited reference range of RT-PCR that did not include these mutations. In the era of precision medicine, the increasing number of molecular alterations that should be tested in clinical practice requires the implementation of highly sensitive and specific assays; thus, the restricted reference range represents a crucial limitation of all the currently available RT-PCR assays, including therascreen^®^ which covers 72% of all *PIK3CA* mutations [10,39]. The advent of the recent therapeutical implications of *PIK3CA* has further pointed out this limitation, despite older studies considering RT-PCR assays as valid options for this molecular test [40]. Interestingly though, the concordance between the two methods increased to 76.9% when the raw RT-PCR data of three discordant cases were visually inspected, confirming the result of NGS. This finding suggests that coupling the automatized analytical workflow with manual data analysis improves the outcomes of RT-PCR. However, it requires trained personnel for data analysis to overcome false negative results, and in those cases, further validation with another technique is warranted. Importantly, when the analytical performance of the two methods was examined in the primary tumor samples, a similar concordance rate (62.5%) was observed.

Our study has some limitations, including the small sample size, the lack of a validation set, and the fact that not all the metastasis samples had available material for being tested with both NGS and RT-PCR. Additionally, considering that primary tumor samples are not always present due to diagnosis at the later stages of the disease, in our study, primary tumors were analyzed in half of the selected cases. The adoption of an RT-PCR assay different from the companion diagnostic test therascreen^®^ *PIK3CA* may not be in line with the approval by the FDA; however, it was chosen due to its slightly broader reference range. Finally, cases in which the RT-PCR assay either failed or reported a potential false negative result were not tested with another assay due to the limited residual DNA.

## 5. Conclusions

Targeted NGS for *PIK3CA* mutational testing on FFPE tumor and metastasis samples from patients with HR+/HER2− metastatic breast cancers is a reliable and robust diagnostic strategy. On the other hand, a careful selection of cases based on the quantity and quality of the DNA allows for solid RT-PCR-based *PIK3CA* testing both in primary tumors and matched metastases. Given that multiplexing assays allow the simultaneous analysis of several clinically actionable biomarkers, they require a relatively low nucleic acid input and generally have wide reference ranges. In the case of available high-quality material, based on the optimization of the laboratory workflow, RT-PCR would allow for short turnaround times and cheaper tests. Our findings should be expanded to larger cohorts of patients and compared with additional NGS and RT-PCR assays to establish tailored diagnostic algorithms and select the most appropriate test on the most appropriate FFPE samples at a single-patient level.

## Figures and Tables

**Table 1:** Clinicopathological features of the patients included in this study according to the diagnosis of the primary tumor. NST, invasive carcinoma of no special type (aka ductal); ER, estrogen receptor; PgR, progesterone receptor; CDK4/6, cyclin-dependent kinase 4 and 6 inhibitors.

	Patients (*n* = 16)
Age at diagnosis, range (median)	32–73 (52.5)
Histology, *n* (%)	
NST	15 (93.7)
Lobular	1 (6.3)
ER, *n* (%)	
Positive	16 (100)
Negative	0
PgR, *n* (%)	
Positive	8 (50.0)
Negative	8 (50.0)
HER2, *n* (%)	
Low	7 (43.8)
Zero	9 (56.2)
Metastatic site, *n* (%)	
Bone (iliac crest)	2 (12.5)
Bone (pelvis)	1 (6.3)
Lymph node	2 (12.5)
Liver	8 (50.0)
Pleura	2 (12.5)
Thoracic wall	1 (6.3)
Therapy with CDK4/6, *n* (%)	
Yes	6 (37.5)
No	10 (62.5)

**Table 2:** Clinicopathological features at a single-case level and time interval between primary tumor and metastasis of the patients with both samples available. All cases were of no special histological subtype.

#Case	Age at Original Diagnosis	Time between Primary Tumor and Metastasis (Months)	Sample Type	ER (%)	PgR (%)	HER2 (%)
PIK_004	26	69	Primary	95	95	1+
			Metastasis	95	0	1+
PIK_007	46	102	Primary	95	70	1+
			Metastasis	90	2	2+ not amplified
PIK_008	45	54	Primary	95	0	1+
			Metastasis	95	0	2+ not amplified
PIK_010	53	126	Primary	95	0	1+
			Metastasis	95	0	2+ not amplified
PIK_012	43	38	Primary	95	20	2+ not amplified
			Metastasis	95	10	1+
PIK_013	44	117	Primary	95	95	0
			Metastasis	80	90	0
PIK_014	41	116	Primary	95	95	1+
			Metastasis	95	0	0
PIK_016	40	127	Primary	95	95	0
			Metastasis	60	0	0

**Table 3:** Summary of the *PIK3CA* mutations detected using NGS on primary tumor and metastasis samples. Mutations are annotated according to both amino acid change and coding. VAF, variant allele frequency; NGS, next-generation sequencing.

Metastases					Primary Tumors			
#Case	[DNA] (ng/µL)	Tumor Cell Content (%)	Mutation	VAF (%)	[DNA] (ng/µL)	Tumor Cell Content (%)	Mutation	VAF (%)
PIK_001	0.26	20	p.E545K/c.1633G>A	3	-	-	-	-
PIK_002	0.84	60	p.H1047R/c.3140A>G	29	-	-	-	-
PIK_003	0.37	60	p.H1047R/c.3140A>G	67	-	-	-	-
PIK_004	0.24	70	p.H1047R/c.3140A>G	38	2.99	60	p.H1047R/c.3140A>G	42
PIK_005	0.44	80	p.N1068Kfs*5/c.3203_3204insA	20	-	-	-	-
PIK_006	0.42	70	p.E545K/c.1633G>A	35	-	-	-	-
PIK_007	0.36	30	p.H1047R/c.3140A>G	10	0.54	20	p.H1047R/c.3140A>G	41
PIK_008	4.06	85	p.H1047R/c.3140A>G	56	0.41	35	p.H1047R/c.3140A>G	22
PIK_009	0.43	70	p.H1047L/c.3140A>T	24	-	-	-	-
PIK_010	0.24	70	p.E545K/c.1633G>A	25	0.66	60	p.E545K/c.1633G>A	49
PIK_011	4.52	60	p.H1047R/c.3140A>G	44	-	-	-	-
PIK_012	0.48	60	p.E542K/c.1624G>A	18	0.31	80	p.E542K/c.1624G>A	4
PIK_013	113.00	80	p.E453K/c.1357G>A	13	3.06	75	p.E453K/c.1357G>A	37
PIK_014	0.42	50	p.N1044K/c.3132T>A	54	1.83	75	p.N1044K/c.3132T>A	37
PIK_015	0.12	90	p.Q546K/c.1636C>A	37	-	-	-	-
PIK_016	0.07	80	p.Q546R/c.1637A>G	33	0.97	40	p.Q546R/c.1637A>G	45

**Table 4:** Summary of the *PIK3CA* mutations detected using RT-PCR on primary tumor and metastasis samples. Mutations are annotated according to the amino acid change; WT, wild-type; ^#^ mutation out of the EasyPGX reference range; * Failed due to low DNA quantity but mutation visible in the individual report.

	Metastases			Primary Tumors		
#Case	[DNA] (ng/µL)	Tumor Cell Content (%)	Mutation	[DNA] (ng/µL)	Tumor Cell Content (%)	Mutation
PIK_001	0.26	20	E545x	-	-	-
PIK_002	0.84	60	H1047x	-	-	-
PIK_003	0.37	60	H1047x	-	-	-
PIK_004	0.26	35	Failed *	9.11	60	H1047x
PIK_005	0.44	20	WT ^#^	-	-	-
PIK_006	-	-	-	-	-	-
PIK_007	0.18	40	Failed *	0.54	20	H1047x
PIK_008	4.06	85	H1047x	0.41	90	H1047x
PIK_009	0.43	70	H1047x	-	-	-
PIK_010	0.24	70	E545x	2.49	30	E545x
PIK_011	-	-	-	-	-	-
PIK_012	0.48	60	E542x	0.47	90	WT
PIK_013	28.00	70	WT ^#^	12.00	70	WT ^#^
PIK_014	-	-	-	7.31	35	WT ^#^
PIK_015	0.12	90	Failed *	-	-	-
PIK_016	0.14	80	WT	0.97	40	Q546x

## Data Availability

The raw data of this study will be made available upon reasonable request to the corresponding author.

## Supplementary Materials

The following supporting information can be downloaded at: https://www.mdpi.com/article/10.3390/cells11223545/s1; Figure S1: Visual representation of the RT-PCR raw data of cases reported as failed by the EasyPGX^®^ analysis software.
