# Supplementary material for: Analytical Performance of Next-Generation Sequencing and RT-PCR on Formalin-Fixed Paraffin-Embedded Tumor Tissues for PIK3CA Testing in HR+/HER2− Breast Cancer
Source: Cells. 2022 Nov 9;11(22):3545. doi: 10.3390/cells11223545 (PMC9688837; doi:10.3390/cells11223545)
Supplement: Supplementary file 1 [file cells-11-03545-s001.zip › cells-1943076-supplementary.pdf]

A

diatech pharmacogenetics	N345x	C420R	E542x	E545x	Q546x	H1047x	G1049x
Case	Result	Result	Result	Result	Result	Result	Result
PIK_007	FAILED	FAILED	FAILED	FAILED	FAILED	FAILED	FAILED

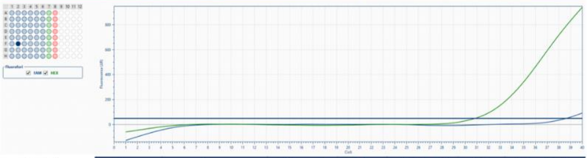

Position	Case	Assay	Channel	Cq	ΔR(Last)	ΔCq	Threshold
F2	PIK_007	PIK3CA H1047x	FAM	38.60	93	6.9	50
F2	PIK_007	PIK3CA H1047x	HEX	30.90	938	-0.5	50

diatech pharmacogenetics	N345x	C420R	E542x	E545x	Q546x	H1047x	G1049x
Case	Result	Result	Result	Result	Result	Result	Result
PIK_004	FAILED	FAILED	FAILED	FAILED	FAILED	FAILED	FAILED

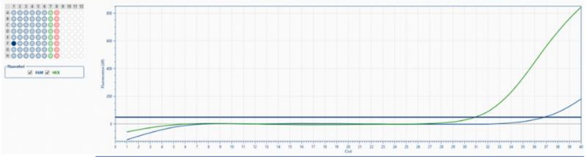

Position	Case	Assay	Channel	Cq	ΔR(Last)	ΔCq	Threshold
F1	PIK_004	PIK3CA H1047x	FAM	36.90	181	5.0	50
F1	PIK_004	PIK3CA H1047x	HEX	30.90	843	-1.3	50

B

diatech pharmacogenetics	N345x	C420R	E542x	E545x	Q546x	H1047x	G1049x
Case	Result	Result	Result	Result	Result	Result	Result
PIK_015	FAILED	FAILED	FAILED	FAILED	FAILED	FAILED	FAILED

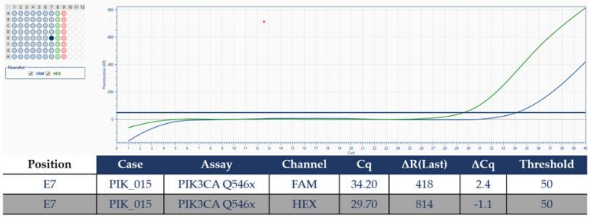

Position	Case	Assay	Channel	Cq	ΔR(Last)	ΔCq	Threshold
E7	PIK_015	PIK3CA Q546x	FAM	34.20	418	2.4	50
E7	PIK_015	PIK3CA Q546x	HEX	29.70	814	-1.1	50

Figure S1. Visual representation of the RT-PCR raw data of cases reported as failed by the EasyPGX® analysis software.
